# Central Nervous System Brucellosis Granuloma and White Matter Disease in Immunocompromised Patient

**DOI:** 10.3201/eid2306.161173

**Published:** 2017-06

**Authors:** Mohammed Alqwaifly, Fahad S. Al-Ajlan, Hindi Al-Hindi, Abdulaziz Al Semari

**Affiliations:** Qassim University College of Medicine, Qassim, Saudi Arabia (M. Alqwaifly);; King Faisal Specialist Hospital and Research Center, Riyadh, Saudi Arabia (F.S. Al-Ajlan, H. Al-Hindi, A. Al Semari)

**Keywords:** central nervous system, brucellosis, neurobrucellosis, epilepsy, refractory epilepsy, Brucella, bacteria, white matter disease, intracranial granuloma, immunocompromised patient, meningitis/encephalitis, zoonoses, Saudi Arabia

## Abstract

Brucellosis is a multisystem zoonotic disease. We report an unusual case of neurobrucellosis with seizures in an immunocompromised patient in Saudi Arabia who underwent renal transplantation. Magnetic resonance imaging of the brain showed diffuse white matter lesions. Serum and cerebrospinal fluid were positive for *Brucella* sp. Granuloma was detected in a brain biopsy specimen.

Human brucellosis is a major zoonotic disease in Saudi Arabia (*1*). This disease is caused by *Brucella* spp., gram-negative bacteria usually transmitted through consumption of raw meat or unpasteurized dairy products (*2*). Brucellosis is endemic to the Arabian Peninsula and countries bordering the Mediterranean Sea (*3*).

Neurobrucellosis occurs in 5%–10% of patients with brucellosis (*4*). The most frequent clinical manifestation is meningoencephalitis (*5*). Mass lesions in the brain are uncommon (*4*). Intracerebral granuloma associated with brucellosis had been reported in a community-acquired infection (*6*). We report an unusual case of neurobrucellosis and seizures in an immunocompromised patient.

## The Study

The patient was a 46-year-old Saudi woman who had chronic hepatitis C, end-stage renal disease of undetermined etiology, and a renal transplant in 1993. She reported a 5-month history of headaches and seizures. Seizures were usually preceded by epigastric pain and a sensation of nausea for few seconds, followed by left arm posturing and loss of consciousness. She did not have fever, weight loss, or joint pain. She lived in a rural area, was involved in animal husbandry, and consumed unpasteurized milk products. Her husband had been treated for brucellosis. Her medications included mycophenolate mofetil (500 mg 2×/d since 1993), prednisone (5 mg 1×/d since 1993), levetiracetam (500 mg 2×/d for 5 mo), and phenytoin (200 mg every night for 1 mo).

Neurologic examination showed left homonymous hemianopia, increased deep tendon reflexes in the left hemibody, and the Babinski sign on the left hallux. Initial laboratory test results, including those for complete blood count, erythrocyte sedimentation rate, C-reactive protein, and liver and renal profiles, were within references ranges. Results of serologic analysis for HIV and hepatitis B virus were negative.

A standard agglutination tube (SAT) test result for *Brucella* spp. was positive (titer 1:320), and a 2-mercaptoethanol test result for *Brucella* spp. agglutination was positive (titer 1:160). An ELISA showed antibodies against *Brucella* spp. in serum (titer 1:5,120). Cerebrospinal fluid (CSF) had a leukocyte count of 21 (90% lymphocytes). Levels of protein, glucose, and lactate dehydrogenase in CSF were within references ranges.

Gram staining of a CSF sample and cultures for bacteria, virus, fungi, and acid-fast bacilli (AFB) showed negative results. Results of PCRs for AFB, cytomegalovirus, and JC polyomavirus were negative.

Serologic analysis of CSF showed *Brucella* IgG (titer <1:20) and antibodies against *Brucella* (titer 1:320). Test results were negative for antibodies against *Aspergillus*, *Aspergillus* galactomannan, *Blastomyces*, *Borrelia*, *Coccidia*, *Cryptococcus*, *Histoplasma*, and *Toxoplasma*.

**Figure 1:**
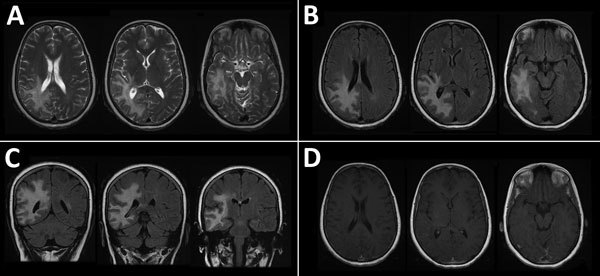
Magnetic resonance imaging of the brain of a 46-year-old immunocompromised woman with central nervous system brucellosis granuloma and white matter disease, Saudi Arabia. A) Axial T2 images showing hyperintensity in the right frontoparietal lobe and right temporal lobe. B) Axial fluid-attenuated inversion recovery (FLAIR) and C) coronal FLAIR images showing that hypersensitivity extends to U-fibers without involvement of the cortex. D) Gadolinium-enhanced image showing that no appreciable mass effect and no central or peripheral enhancement after administration of gadolinium were observed. Each image within each panel shows involvement in different levels of frontal, parietal, and temporal lobes.

An electroencephalogram showed sharp waves over the right temporal region and continuous slow activity over the right temporooccipital region. Magnetic resonance imaging (MRI) of the brain showed diffuse T2/fluid-attenuated inversion recovery hyperintense white matter lesions involving the right frontal, parietal and temporal lobes (Figure 1). No appreciable mass effect or enhancement after administration of gadolinium was observed. Positive emission tomography of the brain showed hypometabolic cerebral activity involving a large area of right cerebral hemisphere. Magnetic resonance spectroscopy shows a low peak of n-acetyl aspartate (2.2 ppm).

**Figure 2:**
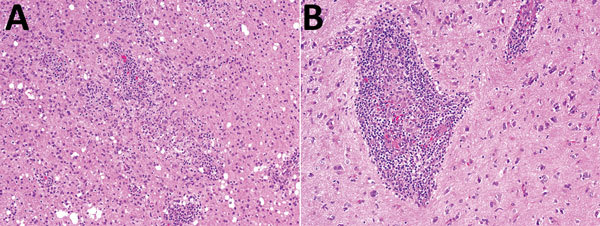
Histologic analysis of a brain biopsy specimen from a 46-year-old immunocompromised woman with central nervous system brucellosis granuloma and white matter disease, Saudi Arabia. A) Low magnification view of cerebral cortex showing infiltration by perivascular lymphocytes and histiocytes. Histiocytes form small nonnecrotizing granuloma (center) (original magnification ×100). B) High magnification view showing an angiocentric epithelioid granuloma cuffed by mature lymphocytes (original magnification ×200). Hemotoxylin and eosin stain.

A brain biopsy specimen of cerebral cortex and superficial white matter showed a moderate lymphoplasmacytic and focally histiocytic infiltrate that involved deep cortex, white matter, and leptomeninges. The histiocytic component formed small epithelioid granulomas that were nonnecrotizing. The inflammatory reaction, including granulomas, was mainly perivascular with some angiocentric patterns and focal parenchymal involvement. The white matter portion was heavily infiltrated by macrophages. Reactive astrogliosis was prominent. There were no morphologic signs of a specific etiology: no viral inclusions, and staining results microorganisms (AFB, fungi, Epstein-Barr virus, and JC polyomavirus) were negative (Figure 2).

A gram stain was initially negative for bacteria. At day 5, *Brucella* spp. were isolated from brain biopsy specimens. An antibiogram showed that the *Brucella* sp. was sensitive to gentamicin, streptomycin, tetracycline, trimethoprim/sulfamethoxazole, and rifampin.

The patient received intravenous ceftriaxone (2 g every 12 h), oral doxycycline (100 mg every 12 h), oral rifampin (600 mg 1×/d), and trimethoprim/sulfamethoxazole (1 tablet [160 mg/800 mg] every 12 h) for 2 wk. After discharge, she was receiving oral doxycycline (100 mg every 12 h), rifampin (600 mg, 1×/d), trimethoprim/sulfamethoxazole (1 tablet every 12 h), and ciprofloxacin (500 mg every 12 h) for 6 mo: she was also receiving levitiracetam (750 mg 2×/d), carbamazepine (200 mg 2×/d), mycophenolate mofetil (500 mg 2×/d), and prednisone (5 mg 1×/d).

Three months later, repeat MRI of the brain showed decreased T2 hyperintensity associated with volume loss and ex vacuo dilatation of the subjacent right lateral ventricle. We did not observe any appreciable new lesions.

After 6 months of follow-up, her headaches had resolved. However, she continued to have auras without major seizures.

## Conclusions

Neurobrucellosis can affect the central or peripheral nervous systems and lead to diverse clinical syndromes (*4*). Diagnosis of neurobrucellosis depends on clinical manifestations, CSF findings suggestive of pinocytosis, high protein levels, low or standard glucose levels, and a positive antibody titer for *Brucella* spp. Although the patient had mild pleocytosis with a predominance of lymphocytes and high antibody titers against *Brucella* spp. in CSF, the CSF protein level was within the reference range. Antibodies against *Brucella* spp. in CSF are usually an indication of neurobrucellosis. However, low levels of antibodies might not be detected by SAT. In suspicious cases in which the SAT result is negative, SAT and a Coombs test, ELISA, and PCR are helpful in making a diagnosis.

77The clinical­−radiologic correlation for neurobrucellosis ranges from uneventful results for imaging studies, despite positive clinical findings, to imaging abnormalities (*3*). Neurobrucellosis with a focal brain mass has been rarely observed in imaging studies (*7*,*8*).

Radiologic results in this case suggested an infectious disease, autoimmune disease, or malignancy in an immunocompromised patient. Because we deemed it necessary to exclude other conditions, such as progressive multifocal leukoencephalopathy or lymphoma, we performed a brain biopsy. The diagnosis was established by detecting antibodies against a *Brucella* sp. in serum and CSF and confirmed by isolation of a *Brucella* sp. from brain tissue.

We found that the patient had epilepsy and extensive white matter changes secondary to brucellosis. She continued to have auras without major seizures. MRI of the brain showed abnormal results (prominent white matter disease and focal encephalomalacia). Inflammation can cause permanent cellular biochemical dysfunction, which can lead to electrically irritable tissue and parenchymal damage despite successful treatment. This finding might explain the persistency of brain lesion. Appropriate antimicrobial therapy can eliminate the infection.

Longitudinal studies of white matter hyperintensities caused by vascular, noninfectious, infectious, and inflammatory conditions showed white matter hyperintensities over time despite effective treatment. Fincham et al. reported that white matter changes in neurobrucellosis were sequelae of demyelination, as confirmed by the pathologic analysis (*9*). We believe that unresolved white matter hyperintensities in this patient were a sequela of the inflammatory process. A case report documented similar clinical features in a patient with seizures caused by chronic neurobrucellosis for 2.5 years (*10*).

Granuloma is a pathogenesis of epilepsy (*11*). Solitary cysticercus granuloma and calcified lesion are 2 common neuroimaging abnormalities in patients with epilepsy. Treatment for underlying cysticercosis does not cure epilepsy (*12*). Seizures associated with central nervous system tuberculomas are often resolved after successful treatment (*13*). The underlying pathogenesis for relapsing epilepsy in neurocysticercosis is probably related to abnormal neurons and their arrangement within calcified nodules (*13*). The epilepsy prognosis for neurobrucellosis is probably similar to that for central nervous system neurocysticercosis (*13*).

A perivascular nonnecrotizing granuloma is a histopathologic feature of neurobrucellosis. Neurocellosis granuloma is a pathogenesis of refractory epilepsy. Our findings indicate the need for suspecting neurobrucellosis as a cause of epilepsy and white matter disease in immunocompromised patients in disease-endemic areas.
